# Antibiotic-Resistant Extended Spectrum ß-Lactamase- and Plasmid-Mediated AmpC-Producing Enterobacteriaceae Isolated from Retail Food Products and the Pearl River in Guangzhou, China

**DOI:** 10.3389/fmicb.2017.00096

**Published:** 2017-02-03

**Authors:** Qinghua Ye, Qingping Wu, Shuhong Zhang, Jumei Zhang, Guangzhu Yang, Huixian Wang, Jiahui Huang, Mongtong Chen, Liang Xue, Juan Wang

**Affiliations:** ^1^Guangdong Institute of Microbiology, State Key Laboratory of Applied Microbiology Southern ChinaGuangzhou, China; ^2^Guangdong Provincial Key Laboratory of Microbial Culture Collection and Application, Guangdong Open Laboratory of Applied MicrobiologyGuangzhou, China; ^3^School of Bioscience and Bioengineering, South China University of TechnologyGuangzhou, China; ^4^College of Food Science, South China Agricultural UniversityGuangzhou, China

**Keywords:** *Enterobacteriaceae*, antimicrobial resistance, ESBL, AmpC, β-lactamase, retail food, water of the pearl river, Guangzhou

## Abstract

We conducted a survey in 2015 to evaluate the presence of extended spectrum β-lactamase (ESBL)- and plasmid-mediated AmpC-producing Enterobacteriaceae in retail food and water of the Pearl River in Guangzhou, China, as well as their antibiotic resistance profiles. Samples (88 fresh food samples and 43 water samples) from eight different districts were analyzed by direct plating and after enrichment. Multidrug-resistant strains were found in 41.7 and 43.4% of food and water samples, respectively. ESBLs were found in 3.4 and 11.6% of food and water samples, respectively, and AmpC producers were found in 13.6 and 16.3% of food and water samples, respectively. Molecular characterization revealed the domination of *bla*_*CTX*−*M*_genes; plasmidic AmpC was of the type DHA-1 both in food and water samples. Thirteen of Fifty one β-lactamase-producing positive isolates were detected to be transconjugants, which readily received the β-lactamase genes conferring resistance to β-lactam antibiotics as well as some non-β-lactam antibiotics. These findings provide evidence that retail food and the river water may be considered as reservoirs for the dissemination of β-lactam antibiotics, and these resistance genes could readily be transmitted to humans through the food chain and water.

## Introduction

Enterobacteriaceae is a large family of gram negative, non-spore forming rods, which are facultative anaerobes and capable of fermenting sugars to various end products. The family Enterobacteriaceae consists of numerous genera of gram-negative bacilli, which include potential pathogens of several genera such as *Escherichia, Enterobacter, Klebsiella, Proteus, Citrobacter, Serratia, Salmonella, Shigella*, and *Yersinia* (Jarzab et al., 2011). These bacteria are transmitted via the fecal material and wastewater in different environments, including soil, water, food, and others (Bain et al., 2014).

Antibiotic resistance is a major public health concern. Because of the wide use of broad-spectrum antibiotics, multidrug-resistant isolates have evolved, and increased in incidence through mutation, selection, and the spread of drug-resistant genes (World Health Organization, 2012). These drug-resistant bacteria can readily be transferred to humans through consumption of contaminated water and food, contributing to the spread and perseverance of antibiotic-resistant bacteria in the general population and environment (Calbo et al., 2011; Leverstein-van Hall et al., 2011; Overdevest et al., 2011). The production of β-lactamase is the primary mechanism of antibiotic resistance in Enterobacteriaceae, and the extended spectrum β-lactamase (ESBL)- and plasmid-mediated cephalosporinase (AmpC)-producing isolates constitute a particularly important branch in this lineage (Bush and Jacoby, 2010; Nordmann et al., 2011). Since the 2000s, ESBL- and AmpC-producing isolates have been reported worldwide. ESBL and AmpC producers have not only been isolated from hospital settings but are also disseminated in other environments such as rivers and foods, domestic, and wild animals, healthy humans, wastewater, and other sources (Ben Sallem et al., 2012; Blaak et al., 2014; Durso and Cook, 2014).

ESBLs have the ability to hydrolyze penicillins, first-, second-, and third-generation cephalosporins, and aztreonam, but not cephamycins and carbapenems, and are usually inhibited by the so-called “classical” β-lactamase inhibitors such as clavulanic acid, tazobactam, and sulbactam, respectively (Lee et al., 2012). Most ESBLs can also confer resistance to fourth-generation cephalosporins (such as cefepime [FEP] or cefpirome) (Bush and Jacoby, 2010). ESBLs include the classical extended-spectrum TEM-, SHV-, and OXA-type enzymes, which have evolved from their parent enzymes (TEM-1, 2, 13; SHV-1; and OXA-1, 2, 10), and also include newer enzymes with a similar spectrum of hydrolytic activity but a different evolutionary history, such as the CTX-M-type enzymes (Livermore, 2008; Pfeiier et al., 2010). SHV- and TEM-type ESBLs were the main cause of third-generation cephalosporin resistance among the Enterobacteriaceae in the 1980s (Pitout, 2012). In the early 2000s, the CTX-M-type ESBLs rose to dominance over the TEM- and SHV-type enzymes (Canton et al., 2012; Peirano et al., 2012). Plasmid-mediated AmpC β-lactamase was first reported in the 1980s (Philippon et al., 2002), which confers bacteria with resistance to a broad spectrum of β-lactams, including penicillins, oxyimino-β-cephalosporins, cephamycins, and (variably) aztreonam (Singhal et al., 2005), but not FEP, cefpirome, and carbapenems; AmpC β-lactamase producers are usually not inhibited by the “classical” β-lactamase inhibitors mentioned above, but are inhibited by boronic acid and cloxacillin (Pitout et al., 2010). Plasmid-mediated AmpC β-lactamases include MOX-, CIT-, DHA-, ACC-, FOX-, and EBC-type enzymes, and CMY-2 of CIT-type enzymes has shown the broadest geographic spread and is one of the main causes of β-lactam resistance at present (Whichard et al., 2005; Egorova et al., 2008).

As the freshwater source to agricultural environments for Guangzhou and surrounding towns, the water of Pearl River has been influenced by non-point and point pollution along with the rapid industrial, agriculture, and municipal development in this region; consequently, the inputs of antibiotics and other contaminants into the river have rapidly increased (Yang et al., 2010, 2011; Li et al., 2011). Previous studies have focused on investigating ESBL and AmpC in clinical settings (Gharout-Sait et al., 2015; Leistner et al., 2015; Nielsen et al., 2015; Soha and Lamiaa, 2015), but few studies have been conducted on the dissemination of ESBL and AmpC in food and agricultural environments (Blaak et al., 2014; Reuland et al., 2014; Veldman et al., 2014). Some reported have described ESBL-producing isolates in chicken, pork, beef, other raw meats (Kant and Mevius, 2013; Ojer-Usoz et al., 2013; Reich et al., 2013; Casella et al., 2015), fresh vegetables (Yuan et al., 2009; Reuland et al., 2014), and even raw milk (Thaker et al., 2012; Abate et al., 2016), Ready-to-eat street-vended food (Campos et al., 2015), but fewer reports have described other types of foods. Moreover, there are limited studies on the presence of antibiotic-resistant Enterobacteriaceae isolates in the aquatic environment and food samples of China (Yuan et al., 2009; Tao et al., 2010), and none of these have focused on the Guangzhou region. Detection of ESBLs and plasmid-mediated AmpC lactamases is necessary for effective surveillance and epidemiology, as well as to develop appropriate infection control strategies associated with resistance mechanisms. Toward this end, the aim of this study was to contribute new knowledge on the diversity of Enterobacteriaceae from retail food and water of Pearl River in Guangzhou, characterize their resistance to antibiotics, and determine the presence of ESBL- and AmpC-producers in the recovered isolates.

## Materials and methods

### Sample collection

During the period from October 2014 to January 2015, a total of 88 fresh foods samples which were composed of raw meat products, aquatic products (including freshwater aquatic product and marine food product), raw vegetables, retail-level ready-to-eat (RTE) food (including cooked meat and roast meat), frozen food (including frozen meat products), and mushrooms, were obtained from eight large supermarkets, and 43 water samples were collected from 16 selected sites of Pearl River in Guangzhou. The sampling sites were distributed throughout eight different districts in Guangzhou: Huadou, Yuexiu, Liwan, Haizhu, Tianhe, Conghua, Panyu, and Nansha. Water samples (225 mL) were collected in sterile bottles at a depth of 50 cm below the water surface in the middle of the river. All samples were maintained below 4°C during transportation, and testing was initiated within 2 h after receipt.

### Isolation and identification of enterobacteriaceae

In brief, 25 g or 225 mL of the respective food or water sample was homogenized in 25 mL Enterobacteria enrichment broth (Huankai, Guangzhou, China) for 30 s in stomacher bags (Huankai, Guangzhou, China). Homogenates were incubated at 37°C for 24 h; thereafter, a loopful of the enrichment broth culture was streaked onto violet red bile glucose agar selective medium (Huankai, Guangzhou, China), and the typical colonies that formed on the selective agar were streaked onto tryptic soy agar (Huankai, Guangzhou, China). Phenotypic characteristics, Gram staining, oxidase, and fermentation tests were performed for the isolated bacterial colonies to screen for the presence of enterobacteria. Gram-negative, oxidase-negative, and fermentation-positive isolates were biochemically identified with API 20E (BioMe′rieux, Marcy I′Etoile, France). Subsequently, the isolates were identified using 16S rRNA gene sequencing in cases in which the API result was not discriminatory. The 16S rRNA gene was amplified under standard polymerase chain reaction (PCR) conditions using the primers 27f (5′-GAGTTTGATYMTGGCTCAG-3′) and 1492r (5′-TACGGYTACCTTGTTACGACT-3′) and sequenced (Lane, 1991).

### Antimicrobial susceptibility testing

The strains were tested for susceptibility to antimicrobial susceptibility using the disk diffusion technique following the protocols of the Clinical and Laboratory Standards Institute Clinical Laboratory Standards Institute (2011). The following 16 AM agents were tested: ampicillin (AMP, 30 μg), amoxicillin/clavulanic (AMC, 20/10 μg), aztreonam (AZM, 30 μg), imipinem (IPM, 10 μg), meropenem (MEM, 10 μg), FEP (30 μg), gentamicin (CN, 10 μg), tobramycin (TM, 10 μg), tetracycline (TE, 30 μg), ciprofloxacin (CIP, 5 μg), levofloxacin (LEV, 5 μg), trimethoprim/sulfamethoxazole (SXT, 1.25/23.75 μg), chloramphenicol (C, 30 μg), cefotaxime (CTX, 30 μg), ceftazidime (CAZ, 30 μg), and cefoxitin (FOX, 30 μg) (all from Oxoid Ltd., Basingstoke, UK). *Staphylococcus aureus* ATCC 25923 and *Escherichia coli* ATCC 25922 were used as quality control strains for this study. Zones of inhibition were measured with a precision caliper to the nearest 0.01 mm. Isolates exhibiting resistance to at least three antimicrobial agents tested were considered as multidrug-resistant strains.

### Phenotypic ESBL/AmpC testing

All enterobacterial isolates were screened for ESBL production by the double-disk synergy test (DDST) using CTX and CAZ with and without clavulanic acid (BD, Franklin Lakes, UN), according to the Clinical Laboratory Standards Institute (2011). Furthermore, a FOX disk (Oxoid, Basingstoke, UK) was added to this test to detect AmpC phenotypes (Stalder et al., 2014). All isolates classified as intermediate or resistant to FOX using CLSI criteria (≤17 mm) were suspected to be AmpC-positive. A confirmation test for AmpC was performed using the three-dimensional extract method (TDEM) according to Coudron et al. (2000) with minor modifications. In brief, 50 μL of a 0.5 McFarland bacterial suspension prepared from an overnight tryptic soy agar (Huankai, Guangzhou, China) was inoculated into 10 ml of tryptic soy broth (Huankai, Guangzhou, China) and the culture was grown for 4 h at 37°C. The cells were concentrated by centrifugation, and crude enzyme preparations were made by freezing-thawing the cell pellets five times. The surface of a Mueller-Hinton agar plate (Huankai, Guangzhou, China) was inoculated with *E. coli* ATCC 25922; a 30 μg cefoxitin disk was placed on the inoculated agar. With a sterile scalpel blade, a slit beginning 5 mm from the edge of the disk was cut in the agar in an outward radial direction. By using a pipet, 25–30 μL of enzyme preparation was dispensed into the slit, beginning near the disk and moving outward. Slit overfill was avoided. The inoculated media were incubated overnight at 37°C. Enhanced growth of the surface organism at the point where the slit intersected the zone of inhibition was considered a positive three-dimensional test result and was interpreted as evidence for the presence of AmpC beta-lactamase. All isolates displaying ESBL or AmpC were further characterized in more detail by resistance gene identification.

### Characterization of β-lactamase genes

The genes encoding TEM, SHV, OXA, and CTX-M enzymes for ESBL, and MOX, CIT, DHA, ACC, EBC, and FOX enzymes for AmpC β-lactamases were analyzed by PCR and sequencing in all ESBL-positive and AmpC-positive isolates. Nucleotides and their deduced amino acid sequences were compared with those included in the GenBank database to confirm the specific type of β-lactamase gene. The primer sequences and their positions, PCR conditions, and references are summarized in Table S1.

### Detection of integrons

The presence of *intI1* and *intI2* genes (encoding class 1 and class 2 integrases, respectively) was examined by PCR (Table S1).

### Conjugation transfer experiments

Conjugation experiments were performed with the plasmid-free recipient strain *Escherichia coli* DH5α (Kallova et al., 1995). In brief, single colonies of the donor and recipient were inoculated in Luria-Bertani broth (Huankai, Guangzhou, China) and grown overnight at 37°C. Subsequently, equal volumes of the donor and recipient cultures were mixed and incubated overnight at 37°C without shaking. Serial dilutions were then plated on Luria-Bertani agar selection plates supplemented with 50 μg/mL ampicillin (National institutes for food and drug control, Beijing, China). Experiments were run in parallel including the donor strain alone and acceptor strain alone as controls for all transconjugation experiments to ensure the effectiveness of the selective plates used. Transconjugants growing on the selection plates were subjected to DDST, TDEM, and PCR to confirm the presence of the ESBL/AmpC phenotype.

## Results

### Identification of enterobacteriaceae isolates

A total of 343 suspicious gram-negative bacterial isolates (248 isolates from food samples and 95 isolates from water samples) were selected from the plates and subjected to biochemical identification. Of these, 282 (82.2%) isolates were confirmed as members of the Enterobacteriaceae family, of which 206 (83.1%) and 95 (80%) strains isolated from food and water, respectively. Among the isolates from retail food, the most abundant genera were *Citrobacter* (17.5% of the isolates belonged to *C. freundii, C. braakii, C. amalonaticus, C. youngae*, and *C. farmeri*), *Klebsiella* (15.0% of the isolates belonged to *K. pneumoniae, K. oxytoca*, and *K. terrigena*), *Morganella* (15.0% of the isolates belonged to *Morganella morganii*) and *Enterobacter* (14.1% of the isolates belonged to *E. cloacae, E. aerogenes, E. amnigenus, E. intermedius, E. asburiae*, and *E. cayogenus*). Species affiliation analysis revealed that the most common taxa were *Morganella morganii* (15.0%), followed by *C. freundii* (13.6%), *E. coli* (8.3%), *E. cloacae* (8.3%), and *K. pneumoniae* (7.8%). The remaining isolates belonged to *Serratia* spp., *Hafnia alvei, Kluyvera* spp., *Proteus* spp., *Pantoea* spp., *Providencia* spp., *Cedecea* spp., *Moellerella wisconsensis, Edwardsiella tarda, Salmonella* spp., *Rahnella aquatilis, Ewingella americana*, and *Buttiauxella agrestis* (Table 1). While among the isolates from water, the most common taxa were *E. coli* (23.7%), followed by *C. freundii* (18.4%), *K. pneumoniae* (17.1%), and *E. cloacae* (15.8%). The remaining isolates belonged to *K. oxytoca, K. terrigena, E. aerogenes, Kluyvera* spp., *S*. *marcescens, S*. *fonticola, P*. *vulgaris, Pantoea* spp., *Morganella morganii*, and *Providencia* rettgeri (Table 1).

**Table 1:** **Percentage of isolated Enterobacteriaceae strains from retail food and water in Guangzhou**.

**Species**	**Isolats from food**	**Isolates from water**	**Species**	**Isolats from food**	**Isolates from water**
*C. freundii*	28 (13.6)	14 (18.4)	*Moellerella wisconsensis*	4 (1.9)	0 (0)
*C. farmeri*	1 (0.5)	0 (0)	*E. intermedius*	2 (1)	0 (0)
*C. brakii*	4 (1.9)	0 (0)	*E. asburiae*	1 (0.5)	0 (0)
*C. yourgae*	1 (0.5)	0 (0)	*E.cloacae*	17 (8.3)	10 (13.2)
*C. amalonaticus*	2 (1.0)	0 (0)	*E. earogens*	5 (2.4)	2 (2.6)
*S. fonticola*	0 (0)	3 (3.9)	*E. cancerogenus*	3 (1.4)	0 (0)
*S. liquefaciens*	1 (0.5)	0 (0)	*E. cayogenus*	1 (0.5)	0 (0)
*S. marsenscens*	9 (4.4)	1 (1.3)	*K. oxoytoca*	12 (5.8)	4 (5.3)
*Kluyvera* spp.	6 (2.9)	4 (5.3)	*K. terrigena*	4 (1.4)	1 (1.3)
*Pantoea* spp.	6 (2.9)	1 (1.3)	*K. pneumoniae*	16 (7.8)	18 (23.7)
*Hafnia alvei*	9 (4.4)	0 (0)	*P. vulgaris*	7 (3.4)	2 (2.6)
*Edwardsiella tarda*	2 (1.0)	0 (0)	*P. mirabillis*	1 (0.5)	0 (0)
*Rahnella aquatilis*	1 (0.5)	0 (0)	*Morganella.Morganii*	31 (15.0)	2 (2.6)
*Cedecea spp*	6 (2.9)	0 (0)	*Salmonella spp*	2 (1.0)	0 (0)
*Ewingella americana*	1 (0.5)	0 (0)	*E.coli*	17 (8.3)	18 (23.7)
*Buttiauxella agrestis*	1 (05)	0 (0)	Total	206 (100)	76 (100)
*Provideneia spp*	6 (2.9)	1 (1.3)			

### Antibiotic resistance patterns

**Figure 1:**
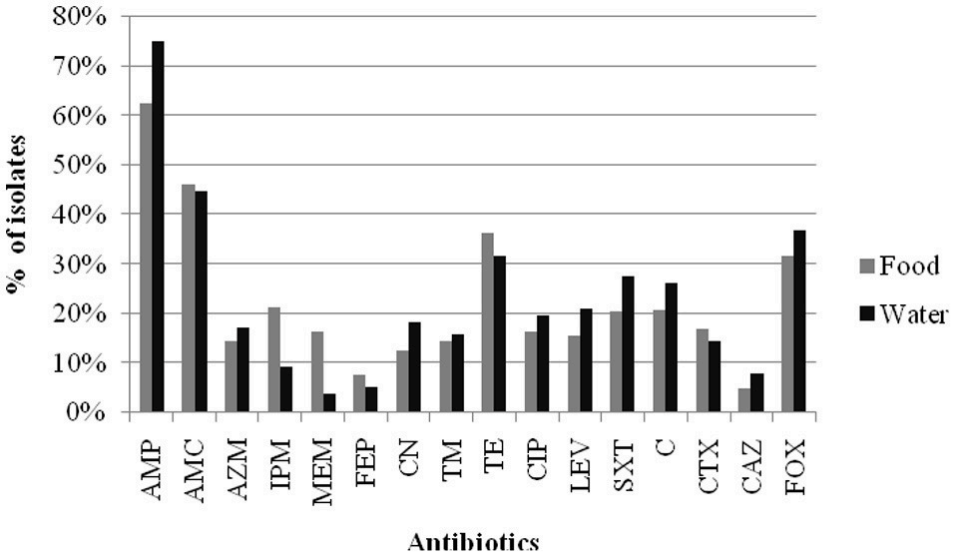
**Results of antibiotic resistance rate of Enterobacteriaceae isolates from retail food and water of the Pearl River in Guangzhou**. Abbreviations: % of isolates were inclued the prevalence of resistance or intermediate resistance to the antibiotics. AMP, ampicillin; AMC, amoxicillin/clavulanic acid; AZM, aztreonam; IPM, imipinem; MEM, meropenem; FEP, cefepime; CN, gentamicin; TM, tobramycin; TE, tetracycline; CIP, ciprofloxacin; LEV, levofloxacin; SXT, trimethoprim/sulfamethoxazole; C, chloramphenicol; CTX, cefotaxime; CAZ, ceftazidime; FOX, cefoxitin.

**Figure 2:**
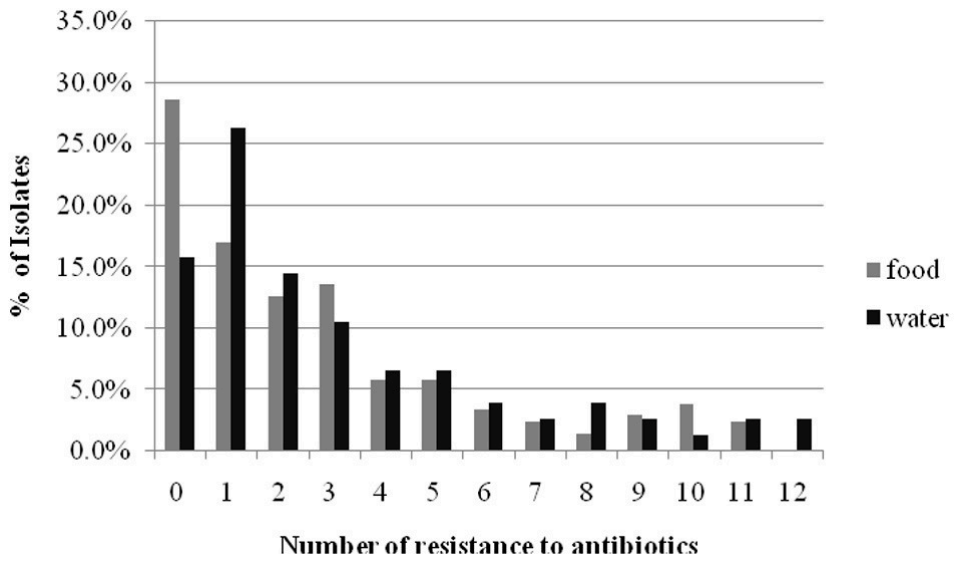
**The comparison of mult-drug resistance rates of Enterobacteriaceae isolated from retail food and water of the Pearl River in Guangzhou**.

The results of the susceptibility testing of the 282 Enterobacteriaceae isolates from food and water against 16 antimicrobial agents are presented in Figure 1. Resistance was observed for all antibiotics tested in this study, and the highest prevalence recorded was against AMP (62.6% in retail food and 75.0% in water). A high percentage of the isolates were resistant to other β-lactam antibiotics tested, such as AMC (46.1% in retail food and 44.7% in water) and FOX (31.6% in retail food and 36.8% in water), but not CAZ (4.9% in retail food and 7.9% in water) and FEP (7.8% in retail food and 5.3% in water). Moreover, high levels of resistance to TE (36.4% in retail food and 31.6% in water), C (20.9% in retail food and 27.3% in water), and SXT (20.4% in retail food and 27.6% in water) were also observed, and the resistance rate to aminoglycoside, quinolones antibiotics varied from 12.6 to 21.1% in retail food and water, respectively. However, approximately 15 and 5% of the strains showed moderate resistance to carbapenems antibiotics in retail food and water, respectively. Approximately 25.2% (71/282) of the isolates were sensitive to all antimicrobials, comprising 28.6% (59/206) and 15.8% (12/76) of those isolated from retail food and water, respectively. In addition, 42.2% (119/282) of the strains exhibited multidrug resistance phenotypes, demonstrating resistance to antibiotics from at least three different classes, including 41.7% (86/206) and 43.4% (33/76) of the strains isolated from retail food and water, respectively (Figure 2).

### Detection of ESBL and AmpC production and characterization of related genes

A total of 53 (18.8%) isolates that showed reduced susceptibility to CTX and/or CAZ by the disk diffusion method were tested for ESBL production. Based on the results, 25 (8.9%) of the isolates were designated as ESBL producers by the DDST, of which 15 (7.3%) and 10 (13.2%) strains isolated from retail food and water, respectively. Genes encoding β-lactamase were detected in these 15 isolates from retail food, mainly in *E. coli* (*n* = 4), *M. morganii* (*n* = 3). The most frequently detected genes were *bla*_*SHV*_ (*n* = 9), followed by *bla*_*TEM*_ (*n* = 4), *bla*_*OXA*_ (*n* = 2), and *bla*_*CTX*−*M*_(*n* = 2). All *bla*_*SHV*_ and *bla*_*TEM*_ sequences were found to encode the broad-spectrum β-lactamases SHV-1 and TEM-1, respectively. SHV-1 was the most dominant genotype (*n* = 9) and was identified in two *E. coli* isolates, and in single isolates of *Serratia liquefaciens, C. amalonaticus, K. oxytoca, Cedecea neteri, M. morganii*, and *E. cloacae*. One isolates carried the *bla*_*SHV*−1_ gene in association with *bla*_*OXA*−1_(*C. frendii*). Four isolates (two *M. morganii*, one each of *E. coli* and *H. alvei*) carried only *bla*_*TEM*−1_. One *E. coli* isolate harbored only the *bla*_*CTX*−*M*−55_ gene, whereas in a *P. mirabilis* isolate, *bla*_*OXA*−1_ was associated with *bla*_*CTX*−*M*−65_ (Table 2). And genes encoding β-lactamase were detected in these 10 isolates from water, mainly in *E. coli* (*n* = 5), *C. frendii* (*n* = 2), and *K. pneumoniae* (*n* = 2). The most frequently detected genes were *bla*_*SHV*_ (*n* = 6), followed by *bla*_*TEM*_ (*n* = 5) and *bla*_*CTX*−*M*_(*n* = 5). All *bla*_*SHV*_ and *bla*_*TEM*_ sequences were found to encode the broad-spectrum β-lactamases SHV-1 and TEM-1, respectively. SHV-1 was identified in single isolates of *E. coli* and *C. frendii*. Three isolates carried the *bla*_*SHV*−1_genein association with *bla*_*CTX*−*M*_(two *E. coli* and one *K. pneumoniae*). One *K. pneumoniae* isolates carried only *bla*_*TEM*−1_, whereas in other isolates, *bla*_*CTX*−*M*−14_ (one *Providencia rettgeri*) and *bla*_*CMY*−2_ (one *E. coli* and one *C. freundii*) was associated with *bla*_*TEM*−1_. One *E. coli* isolate simultaneously harbored the *bla*_*SHV*−1_, *bla*_*TEM*−1_, and *bla*_*CTX*−*M*−65_ genes (Table 3).

**Table 2:** **Results of β-lactamase gene types, integrons and transconjugants of β-lactamase -producing Enterobacteriaceae from retail food in Guangzhou**.

**No**.	**Strains**	**Resources**	**Species**	**β-lactamase phenotypes**	**Integrons**	**Transconjugants^a^**
1	3-3	Raw meat products	*S. liquefaciens*	SHV-1	IntI	−
2	4-2	Raw meat products	*E. coli*	SHV-1	IntI	+
3	19-1	Frozen food	*E. coli*	SHV-1	IntI	−
4	19-4	Frozen food	*E. coli*	CTX-M-55	IntI	+
5	29-2	Raw vegetables	*C. frendii*	SHV-1+OXA-1	IntI	+
6	34-3	Mushroom	*C. amalonaticus*	SHV-1	IntI	−
7	44-2	RTE food	*K. oxytoca*	SHV-1	IntI	−
8	64-1	Raw meat products	*E. coli*	TEM-1	IntI	−
9	51-2	Mushroom	*Cedeca neteric*	SHV-1	IntI	−
10	68B-2	Aquatic products	*Morganella morganii*	SHV-1	IntI	−
11	69-4	Aquatic products	*Morganella morganii*	TEM-1	IntI	−
12	80-3	Aquatic products	*Hafnia alvei*	TEM-1	IntI	+
13	83-2	Raw meat products	*E. cloacae*	SHV-1	IntI	+
14	84-4	Raw meat products	*P. mirabilis*	CTX-M-65+OXA-1	IntI	−
15	88A-2	Aquatic products	*Morganella morganii*	TEM-1	IntI	−
16	1-4	Raw meat products	*C. freundii*	CMY-2	IntI	−
17	10A-4	Aquatic products	*Morganella morganii*	DHA-1	IntI	+
18	10B-1	Aquatic products	*Morganella morganii*	DHA-1	IntI	−
19	13A-2	Aquatic products	*Morganella morganii*	DHA-1	IntI	−
20	13B-1	Aquatic products	*Morganella morganii*	CMY-2	IntI	−
21	15-4	Aquatic products	*E. asburiae*	DHA-1	IntI	−
22	15B-3	Aquatic products	*Morganella morganii*	CMY-2	IntI	−
23	17-3	Frozen food	*C. freundii*	DHA-1	IntI	−
24	45-2	RTE food	*K. oxytoca*	CMY-2+DHA-1	IntI	+
25	72-2	Raw meat products	*Pantoea spp*	ACC-1	IntI	−
26	73-2	Aquatic products	*C. freundii*	DHA-1	IntI	−
27	81-1	Raw meat products	*K. pneumoniae*	DHA-1	IntI	+
28	81-2	Raw meat products	*K. pneumoniae*	DHA-1	IntI	−
29	86A-2	Aquatic products	*Morganella morganii*	DHA-1	IntI	−
30	86-2	Aquatic products	*C. freundii*	DHA-1	IntI	−
31	86-3	Aquatic products	*C. freundii*	DHA-1	IntI	−
32	89-2	Frozen food	*E. cloacae*	DHA-1	IntI	−
33	90-1	RTE food	*Kluyvera spp*	ACC-1	IntI	−
34	96-1	Aquatic products	*Salmonella spp*	ACC-1	IntI	−

a *, +, have been obtained transconjugants; −, have not been obtained transconjugants*.

**Table 3:** **Results of β-lactamase gene types, integrons and transconjugants of β-lactamase -producing Enterobacteriaceae from water of the Pearl River in Guangzhou**.

**No**.	**Strains**	**Resources**	**Species**	**β-lactamase phenotypes**	**Integrons**	**Transconjugants^a^**
1	S1B-1	Water	*E. coli*	CTX-M-65+SHV-1+TEM-1	IntI	−
2	S4-1	Water	*C. frediii*	SHV-1	IntI	+
3	S23A-2	Water	*K. pneumoniae*	TEM-1	IntI	−
4	S17-1	Water	*Providencia rettgeri*	CTX-M-14+TEM-1	IntI	−
5	S19-2	Water	*K. pneumoniae*	CTX-M-14+SHV-1	IntI	+
6	S27-1	Water	*E. coli*	CTX-M-55+SHV-1	IntI	+
7	S37-4	Water	*E. coli*	SHV-1	IntI	−
8	S38-2	Water	*C. freundii*	TEM-1+ CMY-2	IntI	+
9	S40-3	Water	*E. coli*	CTX-M-15+SHV-1	IntI	−
10	S41-1	Water	*E. coli*	TEM-1+CMY-2	IntI	−
11	S16A-1	Water	*S. marcescens*	DHA-1	IntI	−
12	S18-2	Water	*E. coli*	CMY-2	IntI	−
13	S29-1	Water	*E. cloacae*	DHA-1	IntI	+
14	S35-1	Water	*C. freundii*	DHA-1	IntI	−
15	S38-1	Water	*E. cloacae*	DHA-1	IntI	−
16	S41-3	Water	*C. freundii*	DHA-1	IntI	−
17	S42-4	Water	*E. cloacae*	DHA-1	IntI	−

a *, +, have been obtained transconjugants; -, have not been obtained transconjugants*.

A total of 94 (32.9%) isolates that showed reduced susceptibility to FOX were tested for a putative AmpC phenotype. Twenty-eight (9.9%) of the isolates were designated as AmpC producers according to the TDEM, of which 19 (9.2%) and 9 (11.8%) strains isolated from retail food and water, respectively. All 27 AmpC-positive isolates harbored genes encoding AmpCs. Nineteen isolates from retail food were mainly detected in *M. morganii* (*n* = 6), *C. freundii* (*n* = 5), and *K. pneumoniae* (*n* = 2) (Table 2). The most frequently detected genes were *bla*_*DHA*_ (*n* = 13) followed by *bla*_*CIT*_ (*n* = 4), and *bla*_*ACC*_ (*n* = 3), but *bla*_*MOX*_, *bla*_*EBC*_, and *bla*_*FOX*_ were not detected. In all cases, *bla*_*DHA*_, *bla*_*CIT*_, and *bla*_*ACC*_ encoded the AmpC β-lactamases DHA-1, CMY-2, and ACC-1, respectively. Except for one *K. Oxytoca* that simultaneously harbored the *bla*_*CMY*−2_ and *bla*_*DHA*−1_ genes, the others harbored only single AmpC β-lactamase genes (Table 4). Nine isolates from water were mainly detected in *C. freundii* (*n* = 3), *E. cloacae* (*n* = 3), and *E. coli* (*n* = 2). The most frequently detected genes were *bla*_*DHA*_ (*n* = 6) followed by *bla*_*CIT*_ (*n* = 3), but *bla*_*ACC*_, *bla*_*MOX*_, *bla*_*EBC*_, and *bla*_*FOX*_ were not detected. In all cases, *bla*_*DHA*_ and *bla*_*CIT*_ encoded the AmpC β-lactamases DHA-1, CMY-2, respectively. Except for one *K. oxytoca* and one *E. coli* isolate that simultaneously harbored the *bla*_*CMY*−2_ and *bla*_*TEM*−1_ genes, the others harbored only single AmpC β-lactamase genes (Table 3).

**Table 4:** **The occurrence of ESBL- and AmpC producing Enterobacteriaceae isolated from retail food and water of the Pearl River in Guangzhou**.

**Product category**	**Total tests**	**ESBL No. (%)**	**AmpC No. (%)**
Water	43	5 (11.6)	7 (16.3)
Retail food	88	3 (3.4)	12 (13.6)
Total	131	8 (6.1)	19 (14.5)

Overall, 6.1 (8/131) and 14.5% (19/131) of all samples contained ESBL- and AmpC-producing Enterobacteriaceae, respectively. Further subdivision into Enterobacteriaceae species isolated from food and water showed that 11.6 (5/43) and 16.3% (7/43) of the water samples tested, and 3.4 (3/88) and 13.6% (12/88) of the retail food samples tested contained ESBL-producing and AmpC-producing Enterobacteriaceae, respectively (Table 4). Among the retail food samples, ESBL-producing Enterobacteriaceae were detected on one of frozen foods, raw meat products, and raw vegetables respectively, and AmpC-producing Enterobacteriaceae were detected on six of aquatic products, two of frozen foods, two of raw meat products, and two of RTE foods, respectively, with none detected on the mushrooms and vegetables.

**Figure 3:**
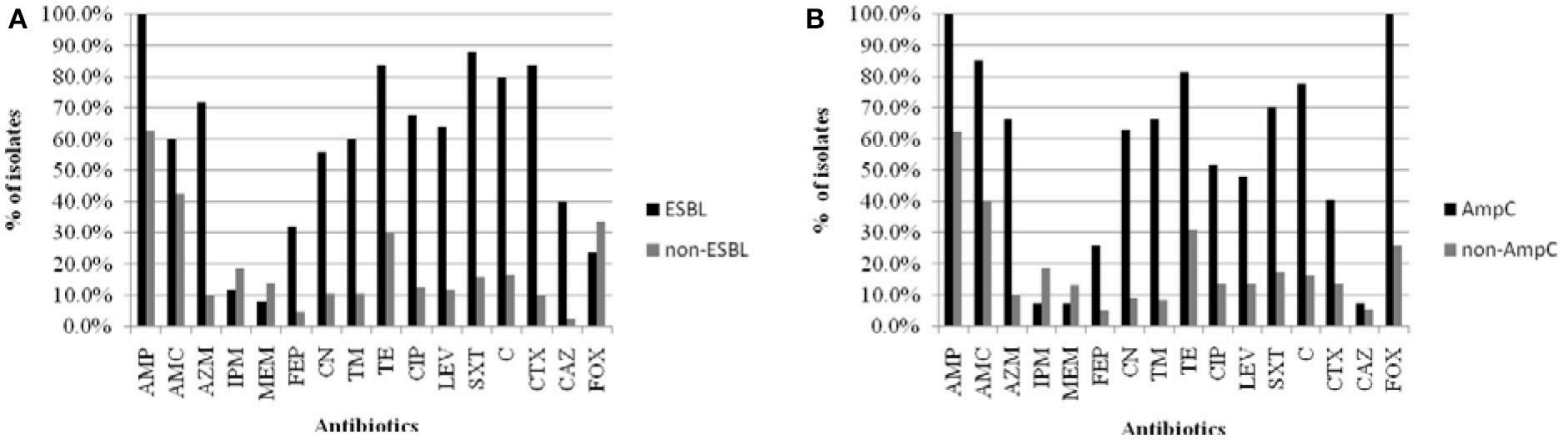
**The comparison of resistant rates between β-lactamase-producing positive strains and negative strains isolated from food and water of the Pearl River in Guangzhou. (A)** ESBL-producing postive strains and negative strains. **(B)** AmpC-producing postive strains and negative strains. Abbreviations: AMP, ampicillin; AMC, amoxicillin/clavulanic acid; AZM, aztreonam; IPM, imipinem; MEM, meropenem; FEP, cefepime; CN, gentamicin; TM, tobramycin; TE, tetracycline; CIP, ciprofloxacin; LEV, levofloxacin; SXT, trimethoprim/sulfamethoxazole; C, chloramphenicol; CTX, cefotaxime; CAZ, ceftazidime; FOX, cefoxitin.

By antibiotic resistance patterns, our study detected a higher resistance rate in β-lactamase-producing positive strains than negative strains (Figure 3).

### Detection of integrons

Class 1 integrons were present in all of the 51 ESBL-(by DDST) and AmpC-producing strains, but class 2 integrons were not identified in this collection (Tables 2, 3).

### Conjugal transfer of ESBL- and AmpC-encoding genes

In the conjugation experiments, transfer of the ESBL and AmpC phenotype was demonstrated in 9 of the 25 ESBL (by DDST) strains tested (5 isolates from food samples and 4 isolates from water samples), and in 5 of 28 AmpC strains tested (3 isolates from food samples and 2 isolates from water samples); however, transconjugants could not be recovered in the other strains. All of the obtained transconjugants received the β-lactamase genes and acquired the ESBL and AmpC phenotypes. In addition, four transconjugants (single *E. coli* and *K. pneumoniae* isolates, and two *C. freundii* isolates) acquired the ESBL and AmpC phenotypes as well as resistance to SXT, TM, TE, and CN, respectively (Table 5).

**Table 5:** **Characterization of transconjugants of β-lactamase -producing Enterobacteriaceae from retail food and water of the Pearl River in Guangzhou**.

**Strains**	**Species**	**Resources**	**Donor (origin)**		**Transconjugants**	
			**β-lactamase phenotypes**	**Antibiotic resistance profiles**	**β-lactamase phenotypes**	**Antibiotic resistance profiles**
4-2	*E. coli*	Raw meat products	SHV-1	AMP-AZM-FEP-TM-TE-LEV-SXT-C-CTX	SHV	AMP-AZM-FEP-SXT-CTX
19-4	*E. coli*	Frozen food	CTX-M-55	AMP-AZM-FEP-CN-TM-TE-LEV-SXT-C-CTX	CTX-M	AMP-AZM-CTX
29-2	*C. frendii*	Raw vegetables	SHV-1+OXA-1	AMP-AMC-AZM-TM-TE-CIP-SXT-C-CTX-FOX	SHV	AMP-AZM-TE-CTX-FOX
80-3	*Hafnia alvei*	Aquatic products	TEM-1	AMP-AZM-CIP-SXT-CTX-CAZ	TEM	AMP-AZM-CTX-CAZ
83-2	*E. cloacae*	Raw meat products	SHV-1	AMP-TM-TE-CIP-LEV-CTX-CAZ	SHV	AMP-CTX-CAZ
10A-4	*Morganella morganii*	Aquatic products	DHA-1	AMP-AMC-IPM-FEP-TE-LEV-SXT-CTX-FOX	DHA	AMP-FEP-CTX-FOX
45-2	*K. oxytoca*	RTE food	CMY-2+DHA	AMP-AMC-CN-TE-CIP-SXT-FOX	CIT+DHA	AMP-FOX
81-1	*K. pneumoniae*	Raw meat products	DHA-1	AMP-AMC-AZM-FEP-CN-TM-CIP-LEV-CTX-FOX	DHA	AMP-AMC-AZM-CTX-FOX
S4-1	*C. frediii*	Water	SHV-1	AMP-AMC-AZM-FEP-CN-TM-TE-CIP-LEV-SXT-CTX-CAZ	SHV	AMP-AZM-CTX-CAZ
S19-2	*K. pneumoniae*	Water	CTX-M-14+SHV-1	AMP-IPM-CN-TM-TE-SXT-C-CTX	CTX-M+SHV	AMP-CN-CTX
S27-1	*E. coli*	Water	CTX-M-55+SHV-1	AMP-AMC-CN-SXT-C-CTX	CTX-M+SHV	AMP-CTX
S38-2	*C. freundii*	Water	TEM-1+CMY-2	AMP-CN-TM-TE-CIP-LEV-SXT-C-CTX-FOX	TEM+CMY+DHA	AMP-TM-CTX-FOX
S29-1	*E. cloacae*	Water	DHA-1	AMP-AMC-TE-LEV-FOX	DHA	AMP-FOX

## Discussion

With the wide use of antibiotics, high levels of antibiotic compounds and antibiotic resistance have been detected in all kinds of environments such as soils, food, water, sediments, and sludge (Knapp et al., 2011; Ben Sallem et al., 2012; Thevenon et al., 2012; Blaak et al., 2014; Durso and Cook, 2014). Therefore, the prevalence of antibiotic-resistant bacteria has become a significant environmental and health challenge. Enterobacteriaceae have been considered to play a major role in the ongoing mobilization of resistance genes from environmental microbes to other species, with eventual transmission to human pathogens, and vice versa (Machado et al., 2009; Tacão et al., 2012). This trend has been mainly addressed through epidemiological studies of the spread of ESBLs in aquatic environments and food-producing animals (Machado et al., 2009; Lu et al., 2010; Tacão et al., 2012). Nevertheless, genes encoding clinically important ESBLs in Enterobacteriaceae besides those in food-producing animals and in the water from Pearl River have not been performed in Guangzhou. Thus, the present study focused on the antibiotic resistance patterns, and identified the ESBL and AmpC producers in retail food and in Pearl River, which is the primary freshwater source for Guangzhou city.

Analysis of the antibiotic susceptibility profile revealed that a very high percentage (43.4%) of the strains isolated from water exhibited a multidrug-resistance phenotype, and the prevalence was higher than that previously reported by others in studies of Pearl River (Tao et al., 2010), and was even higher than the common antibiotic resistance profiles of Enterobacteriaceae in aquatic environments (Zou et al., 2012; Maravic' et al., 2015). In particular, the highest resistance rates were observed against the penicillin antibiotics TE and FOX, which were higher than the common antibiotic resistance profiles of Enterobacteriaceae. Furthermore, the levels of resistance to C, CIP, LEV, and SXT were higher than those previously reported in aquatic environments (Tao et al., 2010). In addition, in the present study, a high percentage (41.7%) of the strains isolated from retail food exhibited a multidrug-resistance phenotype, and the prevalence was higher than that previously reported by others in studies (Fernández-Fuentes et al., 2012; Campos et al., 2015). Notably, resistance to carbapenems has seldom been found, and these drugs therefore remain the first choice for treatment of serious infections for Enterobacteriaceae; however, the prevalence both in retail food and water was higher than that previously reported (Chen et al., 2012). This phenomenon might be explained by the abuse use of carbapenems antibiotics. Therefore, promotion of the rational use of antibiotics should be strengthened, especially the use of carbapenems, and governmental agencies should formulate policies to control the use of antibiotics to help reduce the emergence of resistant strains.

Relatively few publications have described the incidence of ESBL and AmpC producers in Enterobacteriaceae isolated from food, and those that exist have mainly focused on meat products or animals, without discrimination of which species were detected in which food types (Ojer-Usoz et al., 2013; Abdel-Moein and Samir, 2014; Casella et al., 2015; Zurfluh et al., 2015). The present study revealed a prevalence of 3.4% of ESBL-producing Enterobacteriaceae in retail food, which is consistent with previously reported (van Hoek et al., 2015), but lower than detected in other studies (Geser et al., 2012; Ojer-Usoz et al., 2013; Said et al., 2015); while the prevalence of AmpC-producing Enterobacteriaceae is higher than previously reported (van Hoek et al., 2015). In general, a high incidence of ESBL and AmpC producers was detected in foods of animal origin (i.e., raw meat products and aquatic products), which was in agreement with the results of previous studies (Ojer-Usoz et al., 2013; Casella et al., 2015). However, the prevalence of ESBL- and AmpC-producing Enterobacteriaceae in frozen food was higher than that in raw meat products and aquatic products, indicating that frozen foods may be susceptible to cross-contamination in the production process. The present study revealed a prevalence of 11.6% and 16.3% of ESBL- and AmpC-producing Enterobacteriaceae in water samples, which is higher than previously reported (Maravic' et al., 2015). Notably, we found that the prevalence of AmpC- producing bacteria was higher in ESBL- producing bacteria than previously reported (Reich et al., 2013; van Hoek et al., 2015). This phenomenon might be explained by the variable use of antibiotics in different countries.

Throughout the study period, 10 genera of β-lactamase-producing and ESBL-positive Enterobacteriaceae were observed in retail food and water samples. *E. coli* was the predominant ESBL producer both in retail food and water samples, consistent with the results of other studies (Yang et al., 2009; Lu et al., 2010; Mokracka et al., 2012; Korzeniewska and Harnisz, 2013). However, *C. freundii* and *Morganella morganii*, and *E. cloacae* was the most frequently isolated AmpC-producing species in retail food and water samples, respectively, which is in contrast with the predominance of *E. coli* and *K. pneumoniae* in clinical samples (Cejas et al., 2012), as well as the results of previous studies showing the dominance of *Enterobacter* spp. in other environments (Maravic' et al., 2015). The differences in the occurrence of ESBL- and AmpC-producing Enterobacteriaceae in retail food and water may also be associated with the source and quantity of strains, and the variable use of antimicrobial agents in different countries and geographical regions.

Many studies have found that the generation of ESBL and AmpC enzymes is the primary mechanism of antibiotic resistance in Enterobacteriaceae, which allows for the hydrolysis of cephalosporin antibiotics, except for fourth-generation cephalosporins such as FEP (Ben Sallem et al., 2014; Blaak et al., 2014; Durso and Cook, 2014). The present results showed a higher resistance rate in β-lactamase-producing positive strains than negative strains, and all AmpC-producing Enterobacteriaceae strains were resistant to FOX and AMC. Our data suggest that strains harboring plasmid-mediated AmpC β-lactamase have strong ability to hydrolyze FOX, and are usually not inhibited by the “classical” β-lactamase inhibitors such as clavulanic acid, which is consistent with a previous report (Jacoby et al., 2009; Pitout et al., 2010). *In vitro* susceptibility data suggest that the most reliable choice of therapy for infections of ESBL- and AmpC-producing Enterobacteriaceae is FEP and carbapenems (Paterson et al., 2001). In the present study, less than 25% of the β-lactamase-producing strains showed resistance against FEP and carbapenems, which contrasts with previous studies (Chen et al., 2012; Maravic' et al., 2015). This phenomenon might be explained by the variable use of FEP and carbapenems in Guangzhou. The emergence of ESBL- and AmpC-producing strains and the wide use of β-lactam antimicrobial agents has a direct causal relationship; therefore, measures for controlling the abuse of the third generation of cephalosporin should be formulated to reduce the emergence of more and more drug-resistant strains.

Because different countries and regions have different strategies and regulations for antibiotics use, the dominance of ESBL and AmpC enzyme types are varied along with geographical locations. TEM-, SHV-, and CTX-M-type β-lactamases are the major types in Europe and the Americas (Cant et al., 2008), SHV-type β-lactamase is dominant types in Japan (Yagi et al., 2000), and CTX-M -type β-lactamase is predominant types in China (Zhuo et al., 2013). In addition, CTX-M -type β-lactamase is the most prevalent type of ESBL in the world (Ben-Ami et al., 2009; Canton et al., 2012; Peirano et al., 2012). CMY-2 and DHA-1 are widely observed in clinical samples, and CMY-2 is currently the most prevalent type of plasmid-mediated AmpC worldwide (Whichard et al., 2005; Egorova et al., 2008). DHA-1-producing strains have emerged as an important source of infections nationwide in China (Gupta et al., 2003). In our study, SHV- and TEM-type β-lactamases were identified as belonging to the genotypes SHV-1 and TEM-1, respectively. Unlike TEM-1 and SHV-1, which are broad-spectrum β-lactamases that confer resistance to penicillins and first-generation cephalosporins, but not to third- and fourth-generation cephalosporins (Dierikx et al., 2012), CTX-M is derived from the preferential activity for CTX over CAZ (Paterson and Bonomo, 2005; Canton et al., 2012); therefore, the fact that the rate of resistance to CAZ was below that to CTX may be due to the higher prevalence of strains carrying *bla*_*CTX*−*M*_. The CTX-M variants detected in our study (CTX-M-14, CTX-M-15, CTX-M-55, and CTX-M-65) correspond to the most frequently observed CTX-M variants reported in previous studies (Ben Sallem et al., 2012; Reuland et al., 2014; Veldman et al., 2014). Furthermore, we found that DHA-1 was predominant among AmpC-producing Enterobacteriaceae isolates from both retail food and water samples in Guangzhou, which is in agreement with the results of other studies (Gupta et al., 2003). All of the ESBL- and AmpC-producing Enterobacteriaceae detected in the present study showed a multi-resistance phenotype and harbored integrons, which is also consistent with previous reports (Schmiedel et al., 2014; Said et al., 2015). Except for one strain that simultaneously carried both the ESBL and AmpC enzymes, all isolates harbored either ESBL or AmpC alone, demonstrating that the generation of ESBL and plasmid-mediated AmpC β-lactamase is the primary mechanism of antibiotic resistance in Enterobacteriaceae isolated from retail food and water of Pearl River in Guangzhou. In the conjugation transfer experiments, 13 isolates were detected as transconjugants, which illustrated the overall high population of ESBL- and AmpC-producing Enterobacteriaceae in Guangzhou. These transconjugants could receive the β-lactamase genes conferring resistance to β-lactam antibiotics and some non-β-lactam antibiotics. These results indicated that these plasmids not only carry genes encoding ESBL or/and AmpC β-lactamases but can also spread horizontally among Enterobacteriaceae, which has likely contributed to the prevalence of resistance to antimicrobials for β-lactams and some non-β-lactams, leading to a high incidence of multidrug resistance.

## Conclusions

We here report the results of the first investigation of the prevalence of ESBL- and AmpC-producing Enterobacteriaceae isolated from retail foods and water of Pearl River in Guangzhou. This work showed that retail foods (including raw meat products, aquatic products, mushrooms, raw vegetables, and frozen foods) and freshwater sources are important vehicles for the dissemination of ESBL- and AmpC-producing enterobacteria. Therefore, close surveillance of antimicrobial resistance in bacteria from food-producing and derived food products and freshwater should be established as a priority. In addition, strategies to control the use of antimicrobial agents in food and freshwater are urgently required to suppress the release of multiple drug-resistant bacteria harboring ESBL and plasmid-mediated cephalosporinase genes in these reservoirs.

## Author contributions

QY contributed to designed the work that led to the submission, acquired data, and played an important role in interpreting the results; QW contributed to revised the manuscript and approved the final version; GY, HW, and JH helped to acquired data; SZ, JZ, MC, LX, and JW helped perform the analysis with constructive discussions.

## Funding

The authors would like to acknowledge the financial support of National Natural Science Foundation of China (31371780); Science and Technology Program of Guangdong Province (2014B050504007); Science and Technology Program of Guangzhou, China (201508020037); Key projects in the National Science & Technology Pillar Program during the Twelve Five year Plan Period (2013BAD16B05).

### Conflict of interest statement

The authors declare that the research was conducted in the absence of any commercial or financial relationships that could be construed as a potential conflict of interest. The reviewer BJ and handling Editor declared their shared affiliation and the handling Editor states that the process nevertheless met the standards of a fair and objective review.

## Supplementary material

Table S1 **Sequences of primers used in paper**.
