# Supplementary material for: Antibiotic-Resistant Extended Spectrum ß-Lactamase- and Plasmid-Mediated AmpC-Producing Enterobacteriaceae Isolated from Retail Food Products and the Pearl River in Guangzhou, China
Source: Front Microbiol. 2017 Feb 3;8:96. doi: 10.3389/fmicb.2017.00096 (PMC5289952; doi:10.3389/fmicb.2017.00096)
Supplement: Table S1 — Sequences of primers used in paper. [file Table1.docx]

**Supplementary Material**

Table S1 Sequences of primers used in paper.

| Genes | Primer sequence (5’- 3’) | Ampliconsize (bp) | Annealing temperature | Reference |
| --- | --- | --- | --- | --- |
| CTX-M | F: ATGTGCAGYACCAGTAARGTKATGGC | 592 | 55 | Dierikx et al., 2012 |
|  | R: TGGGTRAARTARGTSACCAGAAYSAGCGG |  |  |  |
| SHV | F: TTATCTCCCTGTTAGCCACC | 796 | 55 | Kanamoria et al., 2011 |
|  | R: GATTTGCTGATTTCGCTCGG |  |  |  |
| TEM | F: GCGGAACCCCTATTTG | 964 | 55 | Dierikx et al., 2012 |
|  | R: ACCATTGCTTAATCAGTGAG |  |  |  |
| OXA | F: ACACAATACATATCAACTTCGC | 813 | 61 | Sa´enz et al., 2004 |
|  | R: AGTGTGTTTAGAATGGTGATC |  |  |  |
| MOX | F: GCTGCTCAAGGAGCACAGGAT | 520 | 55 | Pérez-Pérez and Hanson, 2002 |
|  | R: CACATTGACATAGGTGTGGTGC |  |  |  |
| CIT | F: TGGCCAGAACTGACAGGCAAA | 462 | 58 | Pérez-Pérez and Hanson, 2002 |
|  | R: TTTCTCCTGAACGTGGCTGGC |  |  |  |
| DHA | F: AACTTTCACAGGTGTGCTGGGT | 405 | 56 | Pérez-Pérez and Hanson, 2002 |
|  | R: CCGTACGCATACTGGCTTTGC |  |  |  |
| ACC | F: AAC AGC CTC AGC AGC CGG TTA | 346 | 55 | Pérez-Pérez and Hanson, 2002 |
|  | R: TTCGCCGCAATCATCCCTAGC |  |  |  |
| EBC | F: TCGGTAAAGCCGATGTTGCGG | 302 | 58 | Pérez-Pérez and Hanson, 2002 |
|  | R: CTTCCACTGCGGCTGCCAGTT |  |  |  |
| FOX | F: AACATGGGGTATCAGGGAGATG | 190 | 55 | Pérez-Pérez and Hanson, 2002 |
|  | R: CAAAGCGCGTAACCGGATTGG |  |  |  |
| IntⅠ | F: GGGTCAAGGATCTGGATTTCG | 483 | 62 | Sa´enz et al., 2004 |
|  | R: ACATGGGTGTAAATCATCGTC |  |  |  |
| IntⅡ | F: CACGGATATGCGACAAAAAGGT | 788 | 62 | Sa´enz et al., 2004 |
|  | R: GTAGCAAACGAGTGACGAAATG |  |  |  |
